# Delivering precision antimicrobial therapy through closed-loop control systems

**DOI:** 10.1093/jac/dkx458

**Published:** 2017-12-04

**Authors:** T M Rawson, D O’Hare, P Herrero, S Sharma, L S P Moore, E de Barra, J A Roberts, A C Gordon, W Hope, P Georgiou, A E G Cass, A H Holmes

**Affiliations:** 1 National Institute for Health Research Health Protection Research Unit in Healthcare Associated Infections and Antimicrobial Resistance, Imperial College London, Hammersmith Campus, Du Cane Road, London, UK; 2 Department of Bioengineering, Imperial College London, London, UK; 3 Department of Electrical and Electronic Engineering, Imperial College London, South Kensington Campus, London, UK; 4 College of Engineering, Swansea University, Swansea, UK; 5 Imperial College Healthcare NHS Trust, Hammersmith Hospital, Du Cane Road, Acton, UK; 6 University of Queensland Centre for Clinical Research, Faculty of Medicine and Centre for Translational Pharmacodynamics, School of Pharmacy, The University of Queensland, Brisbane, Australia; 7 Royal Brisbane and Women’s Hospital, Brisbane, Australia; 8 Section of Anaesthetics, Pain Medicine & Intensive Care, Imperial College London, London, UK; 9 Department of Molecular and Clinical Pharmacology, University of Liverpool, Liverpool, UK; 10 Department of Chemistry & Institute of Biomedical Engineering, Imperial College London, Kensington Campus, London, UK

## Abstract

Sub-optimal exposure to antimicrobial therapy is associated with poor patient outcomes and the development of antimicrobial resistance. Mechanisms for optimizing the concentration of a drug within the individual patient are under development. However, several barriers remain in realizing true individualization of therapy. These include problems with plasma drug sampling, availability of appropriate assays, and current mechanisms for dose adjustment. Biosensor technology offers a means of providing real-time monitoring of antimicrobials in a minimally invasive fashion. We report the potential for using microneedle biosensor technology as part of closed-loop control systems for the optimization of antimicrobial therapy in individual patients.

## Introduction

Antimicrobial resistance (AMR) threatens to be a leading cause of death by 2050^1^ making it a global patient safety issue. A major driver of AMR is the inappropriate use of antimicrobials in humans and animals.^2^ To date, research in this field has focused on optimizing the selection of antimicrobial agents. However, these strategies often fail to also consider optimization of the dose of the antimicrobial agent, which should aim to be sufficient to maximize bacterial killing whilst negating the harmful consequences of therapy, such as development of AMR and toxicity to the host.

Data are emerging within certain patient populations, such as critically ill patients, describing wide variations in how individuals handle antimicrobials (pharmacokinetics; PK).^3–7^ These wide variations in individual PK appear to be associated with increased variation in the effects of therapy, including outcomes of treatment and the development of AMR (their pharmacodynamics; PD).^3–7^ In response to the observed variations in individual PK, there has been a shift in the focus of therapeutic drug monitoring (TDM) away from primarily being used to prevent toxicity caused by antimicrobials with narrow therapeutic windows, towards enhancing the efficacy of less toxic agents such as the β-lactams, in order to optimize the outcomes of treatment.^4^^,^^8–13^ However, to achieve true individualization of therapy, we require a focus on not just the PK of antimicrobial agents. We must also understand the individual patient’s physiology as well as the characteristics of the organism that we are treating. One method that has been explored widely is the use of Bayesian dose optimization platforms.^3^ Whilst TDM linked with Bayesian forecasting provides a powerful opportunity for delivering individualized care for patients,^3^^,^^14^ several gaps in current strategies for dose optimization of antimicrobials have hindered clinical implementation. Most notably, methods for more-continuous monitoring to allow real-time adaptive dosing of agents are still not available. Other challenges include difficulties in access to appropriate antimicrobial assays,^12^^,^^15–20^ poor integration of dosing software with electronic health records and decision support systems,^3^^,^^21^ challenges with collecting and handling PK samples,^22^^,^^23^ and failures of compliance with PK sampling protocols currently being used by healthcare professionals.^24^

Validation of novel methods for the monitoring and dose optimization of antimicrobial agents is required. Whilst several studies have explored the role of microfluidics,^22^^,^^23^^,^^25^ these are still hindered by many of the problems associated with routine antimicrobial TDM strategies, such as the need for laboratory analysis and transport of blood products. One potential method for avoiding these problems is the development of closed-loop systems based on minimally invasive, microneedle electrochemical sensor technology.^26^ This technology has been demonstrated to be applicable to the management of other conditions, such as diabetes control through individualized insulin delivery^27–31^ and anaesthesia control intra-operatively.^32^^,^^33^ This approach offers a potential avenue for enhancing the precision of antimicrobial therapy across a number of settings where invasive monitoring techniques may not be appropriate, including the community and non-critical care hospital settings. We report the current state of the art within the field of infection that offers a novel approach for the development of closed-loop systems for precision antimicrobial dosing.

## Concept of closed-loop control for individualized antimicrobial therapy

**Figure 1.**
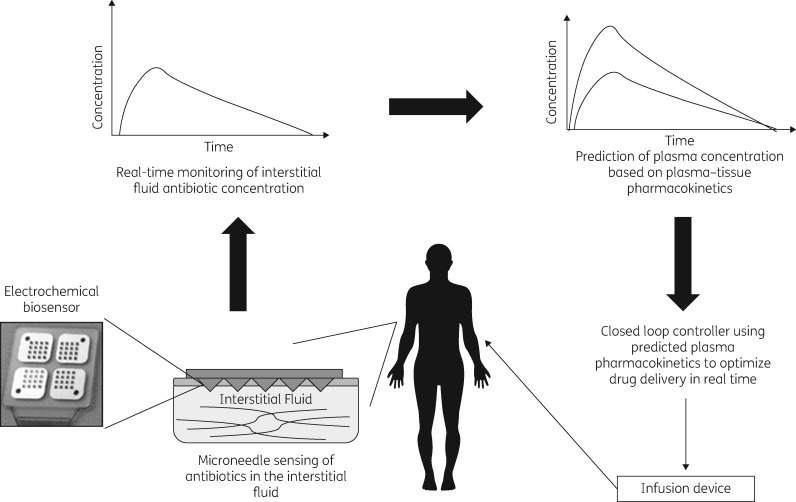
Schematic for closed-loop control of antimicrobial delivery.

There are several key concepts outlined in Figure 1 that must be considered for the development of closed-loop controllers for antimicrobial therapy. Ideally, monitoring of antimicrobials should be continuous and in a minimally invasive format that does not rely on blood sampling. The development of micro-needle array biosensor technology has provided an opportunity to achieve this, allowing for detection of antimicrobial concentrations in the dermal interstitial fluid (ISF).^34^^,^^35^ This technology has already been validated in the field of diabetes, demonstrating safety and tolerability in human clinical trials and accuracy in diabetic individuals who tend to have poor tissue perfusion due to underlying diabetic vasculopathy.^26^^,^^34^^,^^35^ Given that the free antimicrobial concentration in the ISF is generally in equilibrium with the plasma concentration this provides an opportunity for using this technology to monitor ISF concentrations as well as estimate plasma antimicrobial concentration in near real-time without requiring plasma sampling.^36–38^ This may be challenging in certain situations, such as during periods of tissue hypoperfusion in critically ill patients in the intensive care unit (ICU).^39^ However, it may also offer a novel option for supporting the optimization of antimicrobial dosing in these populations. This is because the majority of infections occur in tissue ISF.^39^^,^^40^ Therefore, this technology may provide a mechanism for monitoring antimicrobial concentrations in a compartment that is closer to the site where the infection is being treated when compared with plasma.^39^^,^^40^

Data generated by this sensor can then be linked with machine-driven, closed-loop control algorithms such as Proportional-Integral-Derivative (PID)^41^ and Iterative Learning Controllers (ILC).^42^ These systems will allow for the optimization of both continuous and bolus (or oral) therapy to drive individualized target attainment of pre-defined PK/PD indices associated with maximal bacterial killing and/or suppression of the emergence of AMR.^43^^,^^44^ These may be current gold standard PK/PD targets^45^^,^^46^ or novel indices, such as AUC:EC_50_ ratio.^47^^,^^48^

Each of these concepts will individually be explored and critiqued within this manuscript.

## Microneedles for continuous sensing of agents in the dermal interstitial fluid

Microneedle technology was first demonstrated as a suitable mechanism for drug monitoring and delivery over 20 years ago.^49^ Since then, microneedle technology has progressed rapidly with data supporting the use of microneedles to monitor glucose and lactate concentrations in humans^34^^,^^35^^,^^50^^,^^51^ as well as acting as delivery systems for drugs and vaccines.^30^^,^^52^ Microneedles work by penetrating the stratum corneum layer of the skin allowing access to the ISF, whilst avoiding the nerve fibres and blood vessels that are found within the dermis. Therefore, this offers a minimally invasive method for drug or metabolite monitoring.^34^^,^^35^^,^^50^^,^^51^ Side effects such as pain, bleeding, skin reactions, and infection risk have all previously been explored and shown to be minimal following application of such devices to the skin.^34^

One example of such technology was recently reported by Sharma and colleagues,^28^ who demonstrated high reproducibility when using microneedle technology to monitor glucose levels in healthy volunteers compared with capillary blood glucose measurements. The authors were able to demonstrate robustness of the device to sterilization using gamma-irradiation thus allowing the device to be sterilized and stored over time for use in monitoring human glucose concentrations.^28^ Furthermore, this technology can be reproduced reliably and at low cost through the development of scalable microneedle fabrication batch processing, producing up to 300 microneedles every hour.^50^

However, there are also challenges that remain in the development of microneedles within this field. Whilst microneedle**-**based methods of microdialysis have also been reported for the monitoring of vancomycin,^53^ this technique requires transfer of small volumes of ISF, which not only presents technical challenges in maintaining accuracy of the sensor but also leads to delays that mitigate against their application in real-time control.^53^ Moreover, in clinical trials for monitoring glucose using glucose oxidase-coated microneedles, the sensors appear to occasionally generate artefact during movements that cause them to be partially removed from the intradermal space.^28^ Whilst the artefact present in previous human studies had a shorter duration than changes in glucose concentration, this still requires consideration. Another challenge encountered with current microneedle sensors in humans has been accuracy of these devices at extreme ranges of glucose, especially hypoglycaemic ranges.^28^ It is likely therefore that sensor deployment for antimicrobial monitoring will encounter similar barriers for consideration.

In addition to microneedle-based sensing, other methods to facilitate continuous monitoring are also under consideration. Probably the most developed are attempts to perform real-time monitoring of drug concentrations in ambulatory animals using invasive vascular catheter insertion.^54^ These would only be acceptable in very specific situations in clinical practice, such as critical care or at the time of surgery, where tissue hypoperfusion may influence the ability of microneedle devices to accurately predict free drug concentrations in blood. However, invasive devices pose their own risks to the patient, including thrombosis.^54^ This type of invasive device would not be acceptable in the vast majority of individuals who receive antimicrobial therapy outside of critical care in hospital or in the community settings. A second consideration is the use of non-invasive, sweat-based monitoring systems as have been developed for glucose monitoring. However, to date very few data exist on whether this would be a viable option for monitoring antimicrobial concentrations.^55^

## Antimicrobial electrochemical sensing

Electrochemical sensors for antimicrobials in the environment, agriculture, and humans have been demonstrated for a wide range of agents used in human medicine (Table 1). In the literature, electrochemical sensors for the detection and monitoring of antimicrobials are largely based on aptamer, antibody-linked, or enzyme-based sensors.^54^^,^^56^^,^^57^ These have demonstrated high sensitivity for monitoring of antimicrobials in potentially physiological ranges seen in ISF.^26^ However, there remains a paucity of data for many antimicrobial agents to accurately support the ability of these devices to predict the PK in both tissue and plasma at present. Aptamer sensors are nucleic acid-based and are highly specific for their target molecule, producing their signal through the detection of a redox reaction on ligand binding. Engineered using an *in vitro* selection procedure, called Systematic Evolution of Ligands by EXponential enrichment (SELEX) they have been reported to have a high sensitivity down to the range of picomoles in monitoring of certain environmental contaminants.^56^ One such aptamer-based sensor is the MEDIC device, described by Ferguson and colleagues.^54^ This device has been demonstrated in live animal models to be able to monitor in real time a number of different agents, including kanamycin, using a liquid phase filter to prevent interference from blood-fouling of the DNA aptameric sensor.^54^ Within that study, live rats were injected with increasing doses of kanamycin, an aminoglycoside antibiotic, at hourly intervals to demonstrate the ability to monitor the PK profile in real time using an aptamer sensor in the bloodstream.^54^ Aminoglycoside aptamers have also been tested against spiked human serum demonstrating accuracy for determining concentrations of routine, clinically observed targets between 2 and 6 μM.

**Table 1.** Current antimicrobial sensor classes reported in the literature

Sensor	Setting demonstrated	Ranges of detection in study	Ref
Macrolides	Spiked human urine Water samples Optimal analytical conditions	*In spiked human urine*: 0–2 μM (azithromycin)	^83^^,^^84^
Quinolones	Spiked human plasma Spiked human urine Milk Optimal analytical conditions	In spiked human plasma: 0.05–100 μM (CIP) 0.1–100 μM (OFX) 0.1–40 μM (NOR) 0.06–100 μM (GAT)	^85–90^
Chloramphenicol	Milk Spiked human urine Food samples Optimal analytical conditions	In food samples: 0.08–1392 μM LLD 0.015 μM	^91–94^
Metronidazole	Spiked human urine Optimal analytical conditions	Calibration in lab: Linear range 0.8 pM–720 nM *In spiked urine samples* reported recovery at concentrations 87, 96, 110, and 123 μM	^95^
Tetracyclines	Meat/feedstuff samples Spiked honey Optimal analytical conditions	In feedstuff Linear range 0.3–52.0 μM (tetra) LLD 0.10 μM (tetra)	^96^^,^^97^
Rifampicin	Optimal analytical conditions	Linear detection ranges: 0.006–10.0 mmol/L with an LLD of 4.16 nmol/L and 0.04–10 mmol/L with an LLD of 2.34 nmol/L	^98^
Penicillins	Optimal analytical conditions Food/milk samples	In spiked milk samples: linear range 3–283 μM and LLD 0.3 μM (Pen-G) Recovery from spiked samples was 102±6% In optimal conditions: Km value 67±13 μM reported using Michaelis Menten kinetics equation (Pen-G)	^26^^,^^58^^,^^59^^,^^99–113^
Aminoglycosides	Optimal analytical conditions Ambulatory animals bloodstream Spiked human serum	In spiked human serum: Accurate within therapeutic range of 2–6 μM	^52^^,^^98^^,^^114–118^
Lincomycin	Optimal analytical conditions Foodstuff Spiked human urine	In optimal conditions: Linear detection range up to 1 mM and LLD of 0.08 μM In spiked human urine: Recovery in samples was 96.44% to 103.26%	^119^
Sulphonamides	Optimal analytical conditions Milk Spiked human urine	In optimal conditions: Range of 0.1–10.0 mmol/L with LLD of 60 nmol/L (TMP) AND 1.0–10.0 mmol/L with LLD of 38 nmol/L (SMX) In spiked urine: Recovery 91.3%-101%	^120–122^

CIP, ciprofloxacin; OFX, ofloxacin; GAT, gatifloxacin; NOR, norfloxacin; TMP, trimethoprim; SMX, sulfamethoxazole; Pen-G, penicillin G; LLD, lower limit of detection.

Enzymatic penicillin G sensors are some of the oldest antimicrobial sensors reported in the literature.^57^ These reactions can be detected through electrical, optical, or calorimetric methods.^58^ The majority of these techniques detect the hydrolysis of penicillin to penicillinoic acid and a hydrogen ion. One recent example of this technology is reported by Ro-Lee and colleagues utilizing field effect devices.^59^ The authors describe the high sensitivity of the enzyme-based device, its stability during storage, and re-usability over a 30 day period.^59^

These mechanisms for antimicrobial sensing have so far been demonstrated on microchips, disc electrodes, and nanotubes. This makes the devices small and highly transportable. This technology must now be transferred and tested on microneedle array devices to explore the sensitivity of such systems for real-time antimicrobial monitoring. However, based on current evidence provided by microdialysis of critically ill patients’ tissue ISF concentrations, this approach is a potential avenue for estimation of antimicrobial concentrations and real-time monitoring.^36–38^ Preliminary *in vitro* work exploring the monitoring of β-lactam antibiotics (penicillin G, amoxicillin, and ceftriaxone) in artificial ISF using microneedles has demonstrated such devices provide plausible results.^26^ However, the major gap in the literature supporting translation currently is a paucity of human, *in vivo* studies with such biosensors to demonstrate their resistance to biofouling from proteins such as albumin and immunoglobulins.^60^^,^^61^ Furthermore, there remains limited data on the expected free antibiotic concentrations within the ISF for many antibiotics to predict the characteristics of tissue PK and allow accurate estimates of the linear range of response that such sensors will be required to work in before translation into human studies.

## Closed-loop control for drug delivery

Closed-loop controllers have a broad application in the field of diabetes, being the cornerstone of novel developments, such as the artificial pancreas system.^31^^,^^62^ Furthermore, closed-loop control has been demonstrated as effective in controlling delivery of both intravenous and inhaled anaesthetic agents during surgery.^32^^,^^63^ This technology has been demonstrated in pre-clinical and *in silico* studies to be transferable to optimization of antimicrobial dosing.^54^^,^^63^ Two of the most widely used controllers for continuous and intermittent bolus infusions are the PID and ILC controllers, respectively.^43^^,^^44^ These controllers are algorithms that optimize the delivery of an agent against a pre-determined set point.

### PID control

PID controllers depend on constant monitoring (e.g. every 5 minutes) and can be used to control continuous infusions maintaining drug concentrations at a set target (e.g. either target concentration or PK/PD index). As their name suggests, following data input the PID has three coefficients; the proportional, integral, and derivative. It alters these three coefficients to optimize the response against its target for therapy. The simplicity and robustness of PID algorithms make them extremely suitable for the range of operating conditions found in healthcare. This may be especially useful in critical care, where there is currently a drive towards continuous infusions of β-lactam antimicrobials and nephrotoxic agents, such as vancomycin, to optimize the PK exposure and PD properties.^38^^,^^64–70^ However, where current protocols require sporadic plasma TDM sampling this mechanism offers an opportunity for real-time response to changes in individual patient PK. For example, this would account for variations in PK caused by changes in the patient’s inflammatory response, fluid shifts, augmented renal clearance, and in changing drain outputs in surgical patients that may currently be missed with sporadic TDM sampling.^71–74^

### ILC in closed-loop control

ILC provides the option for optimization of bolus or oral therapy, with data from continuous monitoring being used to optimize the amount, timing, and rate at which a bolus (or oral dose) is delivered. Like PID, ILC algorithms have wide applications but work on the assumption that during repetitive tasks (such as antimicrobial bolus dosing at regular intervals) there will be some level of error in target attainment (e.g. overshoot or undershoot). Therefore, the ILC aims to adjust the input, in this case the bolus dose, to reduce the transient error encountered during routine drug delivery to optimize the accuracy of such systems. This may be more applicable to non-critical care or the community setting (such as outpatient parenteral therapy or oral dosing) and in specialist populations, such as paediatrics and pregnancy, where rich data collection will allow for tailored therapy to be determined and adjusted for, based on real-time data and potentially previous experience housed within machine learning algorithms, as has been demonstrated by the use of Case-Based Reasoning in diabetes management.^75^

These systems can automatically control the delivery of an agent to optimize drug delivery to achieve defined PK/PD targets. If linked with Bayesian dose optimization software or Case-Based Reasoning platforms, which can provide individualized initial dose selection, and novel *in vivo* mechanisms of predicting antimicrobial PD, these could offer a powerful mechanism for reducing the errors that are commonly observed in the practice of current dose optimization strategies.

In terms of translating these into microneedle sensor-driven closed-loop control systems, the biggest challenge remaining is accurately describing the relationship for individual antimicrobials between tissue and plasma PK, especially during the initial phase of dosing, when the drug is not at steady state. This will be required to accurately describe the relationship between free concentrations of drug in both compartments and will likely require rich plasma and microdialysis PK sampling to enable development of accurate algorithms to support such controllers.

## Additional PK/PD indices for individualizing therapy

Currently, individualized PK/PD indices rely on factors such as the MIC to form part of time- and concentration-dependent measures for exposure response (such as AUC:MIC, Time>MIC, or Peak:MIC). MIC as a PD target requires isolation of the causative pathogen and determination of the individual organism’s susceptibility. This causes a practical problem in cases where the invading pathogen is not identified, as is observed during the empirical phase of antimicrobial therapy, and in a significant proportion of cases of sepsis that remain culture-negative throughout the treatment period.^76^^,^^77^ Therefore, in the absence of microbiology results, population-level assumptions are made about the most likely organism causing the infection and the average MIC of this population. Thus this does not provide a truly individualized index on which to optimize antimicrobial therapy.

Furthermore, in place of an easily available individualized PK/PD index to guide the assessment of response to therapy, clinicians rely on clinical judgement, physiological parameters, and biochemical markers such as C-reactive protein (CRP) and procalcitonin (PCT) to assess individual patient response.^78^^,^^79^ In particular, CRP, an acute phase protein that is a non-specific marker of inflammation, is one of the most commonly used biomarkers during infection management in clinical practice.^80–82^ Despite its wide use in infection management, very little attempt has been made to link it directly to exposure–response using PK/PD modelling.

To address this, recent studies have reported the use of the ratio of the AUC to the EC_50_ in paediatric populations.^47^^,^^48^ The EC_50_ is the concentration of a drug (mg/L) that is estimated to induce a half-maximal antibacterial effect (such as reduction in serum CRP or galactomannan, a specific plasma marker in *Aspergillus* infection) for an individual patient. The AUC:EC_50_ ratio can provide an *in vivo* estimate of drug response by linking drug exposure with PD.^47^^,^^48^ Acting as an *in vivo* measure of potency, AUC:EC_50_ enables an estimate of the host immune response to the invading organism. This has the potential to circumvent the problems associated with *in vitro* MIC estimation and may provide data that can drive the development of real-time algorithms for the delivery and control of individualized antimicrobial therapy. With the clinical validation of tools such as the AUC:EC_50_ for predicting antimicrobial PD in individuals using markers such as CRP, future work must now explore the role of using newer infection-related biomarkers, such as procalcitonin and CD64 for improving the accuracy of these tools. Furthermore, exploration of similar methods for predicting toxicity (e.g. renal toxicity) may further enhance the individualization of therapy by including host, antimicrobial agent, and pathogen factors in estimations of the outcome of therapy.

## Drug delivery

Whilst intravenous and oral delivery of agents, via infusion pump and personalized dosing alerts respectively, may be the initial routes for antimicrobial delivery using such control systems there is also the potential for delivery via microneedle systems in the future. Such microneedles are now under investigation for drug and vaccine delivery that provide dual functions of sensing and also drug delivery.^52^ However, in the field of infection, the rate of drug delivery that can be achieved may be hindered by certain drug characteristics (such as hydrophilic versus hydrophobic agents) and the volume of agent required to be delivered. However, this technology may pose an interesting avenue for certain challenging cohorts, such as paediatric patients, as well as for local antimicrobial therapy delivery, such as skin and soft tissue infections or penetration of collections.

## Conclusions

Novel systems are urgently required to individualize delivery of antimicrobial therapy, to address the wide variations in PK currently observed across a range of patient populations, and minimize the impact of sub-optimal dosing on clinical outcomes and AMR. Closed-loop control utilizing dermal antimicrobial sensing techniques offers a potential new avenue of applied research that addresses many of the current barriers associated with drug monitoring and dose optimization tools. Furthermore, the nature of minimally invasive sensor technology provides a platform that can be used across a range of settings from the community to those in intensive care. To achieve this there must be cross-disciplinary collaboration to explore the utility of such technologies to optimize the precision of antimicrobial therapy by addressing a number of the hurdles that remain to implementing this type of technology.
